# Immune thrombocytopenia in a 68‐year‐old woman after COVID‐19 vaccination

**DOI:** 10.1002/ccr3.4689

**Published:** 2021-08-24

**Authors:** Ashley Kenney, Anju Adhikari

**Affiliations:** ^1^ Ascension Columbia St. Mary’s Milwaukee Hospital Milwaukee WI USA

**Keywords:** acute medicine, haematology, infectious diseases, patient education, social care

## Abstract

This case discusses a patient who presented with immune thrombocytopenia purpura (ITP) one week after COVID‐19 vaccination, introducing ITP as a consideration for a potential, but rare, adverse reaction to COVID‐19 vaccination.

## Graphical abstract

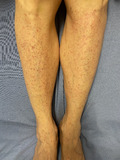

## 1 INTRODUCTION

A 68‐year‐old woman presented with thrombocytopenia, petechial rash, and minor gum bleeding 7 days after Moderna mRNA‐1273 COVID‐19 vaccination. Clinical presentation and laboratory findings were consistent with immune thrombocytopenic purpura (ITP); therefore, the diagnosis of ITP was made and treated.

Moderna mRNA‐1273 vaccine is a nucleoside messenger RNA‐based vaccine that received Emergency Use Authorization for prevention of COVID‐19 by the FDA on December 18, 2020. There were no hematologist side effects reported in the phase 3 trial of the vaccine. However, cases of thrombocytopenia following mRNA‐based COVID‐19 vaccines have been reported through the press and are currently being studied. We present a case of immune thrombocytopenia purpura (ITP), an acquired thrombocytopenia caused by autoantibodies against platelet antigens with an incidence of about 3.3 per 100,000 adults per year,^1^ that developed in a patient one week following Moderna COVID‐19 vaccine. This case introduces ITP as a consideration for a potential, but rare, adverse reaction to COVID‐19 vaccination.

## 2 CASE REPORT

A 68‐year‐old woman with past medical history of hypothyroidism, primary hyperaldosteronism, osteoporosis, and migraine headache disorder received dose 1 of 2 of the Moderna mRNA‐1273 COVID‐19 vaccination in 2021. Seven days post‐vaccination, the patient experienced a widespread petechial rash, minor bruising, and gum bleeding. These symptoms prompted her visit to a local urgent care center, where she was found on laboratory testing to have thrombocytopenia with a platelet count of 15 × 10^9^/L. She was immediately referred to a local emergency department where repeat testing was performed, and results demonstrated an even more profound thrombocytopenia with a platelet count of 4 × 10^9^/L.

The patient was up to date on vaccinations, including yearly influenza, with no history of adverse reactions. Patient had no other reported side effects or symptoms after her vaccination other than minor injection site myalgia for about 1 day. She also has no prior history of allergies or adverse reactions to medications. No new changes or additions had recently been made to her medication regimen which included levothyroxine, potassium chloride, calcium and vitamin D supplements, raloxifene, spironolactone, and sumatriptan. She denied any recent illnesses or being around anyone that was ill. She denied respiratory, cardiac, gastrointestinal, and urinary symptoms. She had no personal or family history of bleeding disorders or platelet disorders.

**FIGURE 1:**
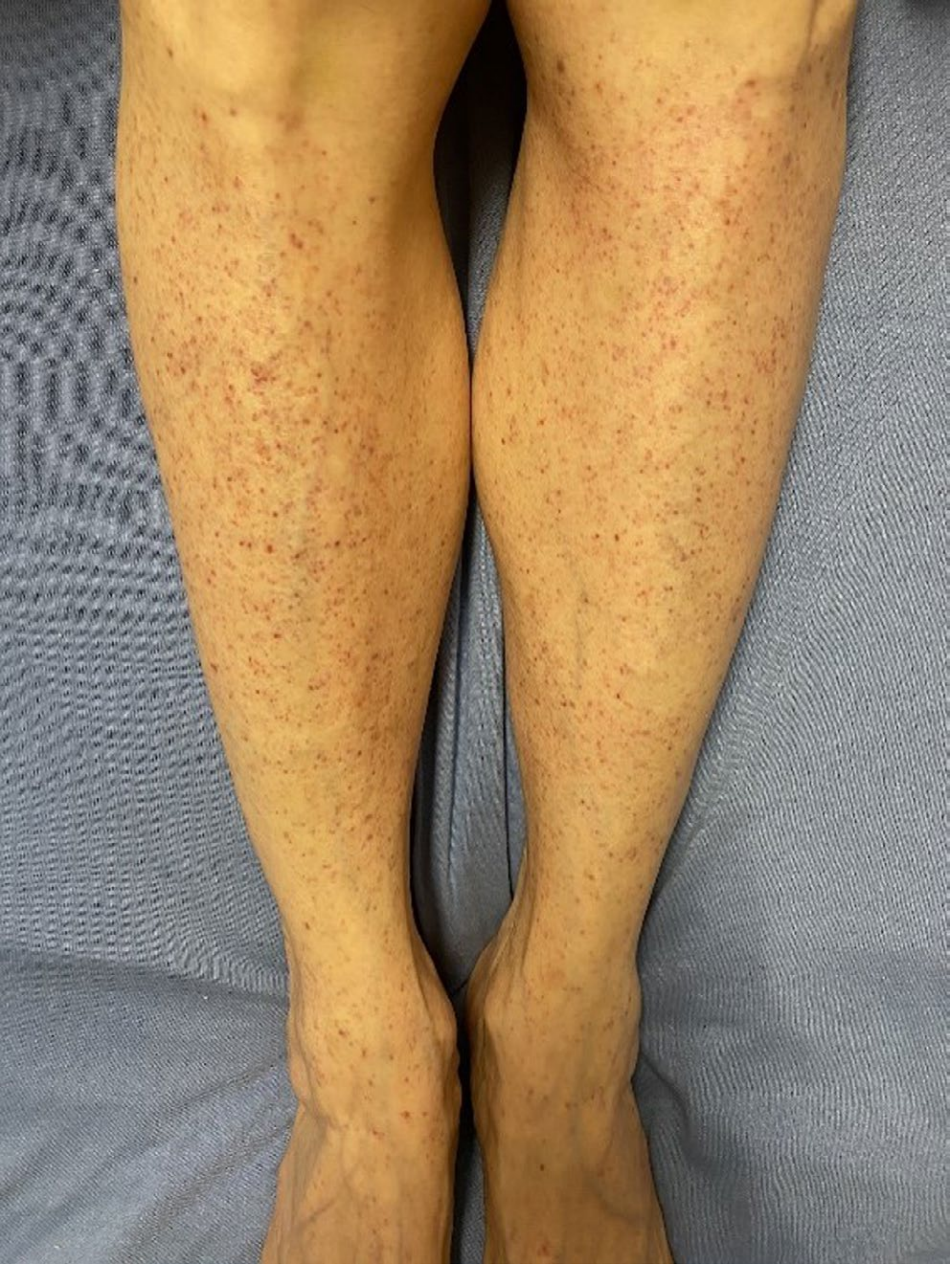
Petechial rash on bilateral lower extremities

On examination, the patient had a diffuse petechial rash most notably on bilateral lower legs but also present on bilateral arms, chest, forehead, and hard palate (Figure 1). There were small hemorrhagic vesicles located on the patient's fingers and on the lateral aspect of her tongue. Additionally, fresh ecchymoses on the patient's anterior legs were noted on the patient's integument examination. The patient's vital signs and remainder of her physical examination were unremarkable. Of note, there was no evidence of splenomegaly.

Laboratory results included white blood cell count 9.7 × 10^9^/L, hemoglobin 119 g/L (11.9 g/dl), hematocrit 0.34 L/L (34.1%), and platelet count 4 × 10^9^/L. Other laboratory testing that were all within normal limits included electrolytes, liver function tests, estimated GFR, total protein, and albumin. Hemolytic markers such as bilirubin and haptoglobin were within normal limits. Peripheral blood smear showed normal morphology of blood cells and no schistocytes. Clinical presentation and laboratory findings were consistent with immune thrombocytopenic purpura (ITP); therefore, the diagnosis of ITP was made. Secondary causes of ITP were investigated. The patient tested negative for HIV 1, HIV 2, hepatitis B virus, hepatitis C virus, and Epstein‐Barr virus serology was negative for acute infection. Patient had no history of joint pain or swelling, rashes, fatigue, and fever, suggestive of rheumatological disease so no further autoimmune laboratory workup was investigated.

## 3 OUTCOME AND FOLLOW UP

The patient was admitted to the hospital and was given intravenous immunoglobulin (IVIG) at 1 g/kg for 2 doses over 2 days as well as started on 60 mg oral prednisone daily for ITP. On day 2 of hospitalization, after the first dose of IVIG and prednisone were given, the patient's platelet count increased to 14 × 10^9^/L. On day 3 of hospitalization, after the second doses of IVIG and prednisone were given, the patient's platelet count significantly increased to 88 × 10^9^/L. IVIG was stopped at that point and she was discharged home with a 60 mg prednisone dose per day with a plan to taper over 3–4 weeks. The patient had a follow‐up appointment with her primary care provider as well as the hematology clinic one week after hospital discharge and at that time, her platelet count had normalized to 258 × 10^9^/L.

## 4 DISCUSSION

ITP has been reported in several patients with COVID‐19 infection.^2^, ^3^ Although the current incidence rate of ITP associated with a COVID‐19 diagnosis is currently unknown, there have been at least 45 cases documented in medical literature.^4^ The leading theory to explain the pathogenesis of these cases of ITP is that viral antigens cross‐react with normal platelet antigens in molecular mimicry, causing dangerous platelet destruction.^3^ Other viruses that are well known to cause this phenomenon include varicella‐zoster virus, HIV, and hepatitis C virus.^3^ ITP has also been associated with other vaccinations.^5^ Autoimmunity after vaccine administration is believed to be caused mostly by viral antigens and less likely by other vaccine constituents.^5^

Outside of reports in the press,^6^, ^7^, ^8^ to the extent of our current knowledge, there has been only one other identified case published in the medical literature of an individual experiencing ITP after a COVID‐19 vaccination.^9^ However, this other individual reported had received the Pfizer‐BioNTech BNT16B2b2 vaccine rather than the Moderna mRNA‐1273 vaccine. In both patients, they had no other associated illnesses or identified cause of ITP and shared the common factor of having been vaccinated against COVID‐19 within 1 week of the onset of ITP symptoms.

As of the writing of this report, over 225 million people in the United States have received at least one dose of a COVID‐19 vaccination.^10^ According to a *New York Times* publication, 36 cases of suspected ITP after COVID‐19 vaccination have been reported to the government’s Vaccine Adverse Event Reporting System (VAERS) by the end of January 2021.^7^ It should also be noted that the incidence of ITP is approximately 3.3 per 100,000 adults per year^1^ and the temporal relationship between this patient’s diagnosis and vaccination could have also have been coincidental. However, it is also valuable to consider that other vaccines, most notably the measles, mumps, rubella (MMR) vaccine, have been studied for possible increased incidence of ITP in certain populations, with one study finding a sixfold increase in risk of ITP in 13‐ to 24‐month‐old children during the 6 weeks following MMR vaccination.^11^

## 5 CONCLUSION

Several cases of ITP have been reported with COVID‐19 infection and including our case, at least two vaccine‐related ITP cases have been reported. While the pathogenesis is not completely understood, it can be hypothesized that viral antigens produced by the vaccine could induce immune reactions similar to that caused by the virus. The severity of thrombocytopenia that was presumed to be caused by the vaccine was comparable to that reportedly caused by COVID‐19 infection. This patient was effectively treated with IVIG 1 mg/kg for two total doses and corticosteroids, prednisone 60 mg per day.

This patient's case was reported through VAERS, and the patient gave consent to share this case. This case is valuable to consider regarding continued vaccine post‐approval safety monitoring. It is in the authors’ opinion that potential and extraordinarily rare adverse effects from vaccinations are important to report and study; however, they do not diminish the immense public and individual protection and benefit the COVID‐19 vaccines provide against a life‐threatening virus.

## 6 PATIENT CONSENT STATEMENT

The patient's permission was granted to share this case as well as images.

## CONFLICT OF INTEREST

The named authors have no conflict of interest, financial or otherwise.

## AUTHOR CONTRIBUTION

Both authors cared for this patient described in this case as well as contributed to conception of article. AK drafted the case report and AA provided critical revision of the case report. Both authors gave final approval of the final published version.

## Data Availability

Data sharing not applicable—no new data generated.

## References

[1] Terrell DR, Beebe LA, Vesely SK, Neas BR, Segal JB, George JN. The incidence of immune thrombocytopenic purpura in children and adults: a critical review of published reports. Am J Hematol. 2010;85(3):174‐180. doi:10.1002/ajh.21616 20131303

[2] Bennett J, Brown C, Rouse M, Hoffmann M, Ye Z. Immune thrombocytopenia purpura secondary to COVID‐19. Cureus. 2020;12(7):e9083. doi:10.7759/cureus.9083 32676257 PMC7362597

[3] Lévesque V, Millaire É, Corsilli D, Rioux‐Massé B, Carrier FM. Severe immune thrombocytopenic purpura in critical COVID‐19. Int J Hematol. 2020;112(5):746‐750. doi:10.1007/s12185-020-02931-9 32613314 PMC7327458

[4] Bhattacharjee S, Banerjee M. Immune thrombocytopenia secondary to COVID‐19: a systematic review. SN Compr Clin Med. 2020;19(2020):1‐11. doi:10.1007/s42399-020-00521-8 PMC7501509 32984764

[5] Perricone C, Ceccarelli F, Nesher G, et al. Immune thrombocytopenic purpura (ITP) associated with vaccinations: a review of reported cases. Immunol Res. 2014;60(2–3):226‐235. doi:10.1007/s12026-014-8597-x 25427992

[6] Grady D, Mazzei P. Doctor's death after COVID vaccine is being investigated. The New York Times. https://www.nytimes.com/2021/01/12/health/covid‐vaccine‐death.html Published January 12, 2021. Accessed February 24, 2021.

[7] Weintraub K. Death of Florida doctor after receiving COVID‐19 vaccine under investigation. USA Today. https://www.usatoday.com/story/news/health/2021/01/06/death‐florida‐doctor‐following‐pfizer‐covid‐19‐vaccine‐under‐investigation‐gregory‐michael/6574414002/ Published January 10, 2021. Accessed February 24, 2021.

[8] Grady D. A few COVID vaccine recipients developed a rare blood disorder. The New York Times. https://www.nytimes.com/2021/02/08/health/immune‐thrombocytopenia‐covid‐vaccine‐blood.html Published February 8, 2021. Accessed February 24, 2021.

[9] Tarawneh O, Tarawneh H. Immune thrombocytopenia in a 22‐year‐old post Covid‐19 vaccine. Published Jan 21, 2021. Am J Hematol. 2021;96(5):E133–E134. doi: 10.1002/ajh.26106 33476455 PMC8014773

[10] COVID‐19 Vaccines. Centers for Disease Control and Prevention. https://www.cdc.gov/coronavirus/2019‐ncov/vaccines/index.html Accessed March 11, 2021.

[11] Black C, Kaye JA, Jick H. MMR vaccine and idiopathic thrombocytopenic purpura. Br J Clin Pharmacol. 2003;55(1):107‐111. doi:10.1046/j.1365-2125.2003.01790.x 12534647 PMC1884189

